# Current Advancements in the Diagnosis and Treatment of Metastatic Spinal Cord Compression and Its Postintervention Care: A Comprehensive Review

**DOI:** 10.7759/cureus.86049

**Published:** 2025-06-15

**Authors:** Avikalp Kishore, Alessio Piccioni, Ivana L Fiallos Vinueza, Ahmed Rasool Younus Khan, Moutasm Kasab, Deepika Lakshminarasimhan, Duaa Behbehani, Jahed Bushnaq, Olamide Ogunfunwa, Naga V Bittu

**Affiliations:** 1 Medicine, Gloucestershire NHS Foundation Trust, Gloucester, GBR; 2 Medicine and Surgery, Università di studi di Roma “La Sapienza”, Rome, ITA; 3 Medicine, Università Vita Salute San Raffaele, Milan, ITA; 4 Medicine, University of Dundee, Dundee, GBR; 5 Medicine, Istinye University, Istanbul, TUR; 6 Medicine, All American Institute of Medical Sciences, Black River, JAM; 7 Internal Medicine, East Lancashire Hospitals NHS Trust, Blackburn, GBR; 8 Internal Medicine, Washington University of Health and Science, San Pedro, BLZ; 9 Internal Medicine, Federal Medical Center Abeokuta, Abeokuta, NGA; 10 Medicine, Dr NTR University of Health Sciences, Hyderabad, IND

**Keywords:** diagnosis, malignant spinal cord compression, metastatic spinal cord compression, multidisciplinary approach, radiotherapy, steroids

## Abstract

Malignant spinal cord compression (MSCC) is a serious oncological emergency that can result in neurological impairment and significant pain. It is associated with high morbidity, mortality, and a decrease in quality of life. This review reviews the current literature concerning diagnosis, guidelines, timely intervention, and management options in oncology patients with MSCC. A literature search of PubMed using relevant keywords and MeSH terms yielded 604 articles. Of these, 31 studies met the predefined inclusion criteria and were selected for detailed data extraction and synthesis into a narrative review.

MRI remains the gold standard for diagnosis, with newer systems like short tau inversion recovery (STIR) sequences being used, and includes the Bilsky grading system to improve classification and diagnosis for treatment. Radiotherapy and surgery continue to be the main treatment options. A multidisciplinary, personalized approach improves the amount of function recovered, pain control, and quality of life. New advances include robotic-assisted surgery and immunotherapy as future management options. Early recognition and clear, evidence-based guidelines are essential to reduce the morbidity and mortality of MSCC. This includes immediate administration of steroids, MRI imaging, and timely referral to appropriate departments, including acute oncology and neurosurgery. Additional research is needed to determine the main factors that impact patient recovery and quality of life.

## Introduction and background

Introduction

Metastatic spinal cord compression (MSCC) is a debilitating condition that can have significant consequences for patients and is often associated with neurological deficits, severe pain, substantial morbidity, mortality, and drastic decline in quality of life [1]. This complication affects up to 3% to 5% of US cancer patients as per the CDC, and the median survival level for these patients is estimated to be 10 months [2, 3].

Patients suffering from breast, lung, prostate, and, less frequently, kidney cancer are more prone to develop metastatic lesions in the spinal cord due to tumor infiltration. The invasion and establishment of the tumor in the nervous system can involve the arterial system or the intervertebral foramina [4], where the thoracic segments are preferred in comparison to the lumbar and cervical segments [5]. Consequences for underdiagnosis or late detection include vertebral collapse and irreversible neurological injuries [3]

While there has long been consensus on the importance of diagnosing and treating MSCC early, the condition itself continues to be underdiagnosed in its early stages, leading to delays in its treatment and poorer patient outcomes [6]. Patients suffering from MSCC often deteriorate within days, thus representing an emergency [7]. The diagnostic methods available range from clinical clues, laboratory exams, and imaging tools like MRI, CT, and PET-CT. Treatment choices, whether invasive or non-invasive, depend on case and patient-specific factors [8] and aim to improve the patient’s quality of life (QoL) and prevent further deterioration.

This narrative review aims to evaluate the current advancements in the diagnosis and treatment of MSCC and its post-intervention care. To maintain specificity, this review will focus on MSCC in adult patients only and exclude discussions on spinal cord compressions caused by primary tumors, non-cancerous bone diseases, and spinal cord compressions in pregnant patients and pediatric patients, as patients with these conditions have specific prognostic and treatment considerations that cannot be applied to the general populace. The pathophysiology of MSCC will be mentioned only to provide relevant context, with primary emphasis placed on clinical advancements.

The narrative review synthesizes findings from 111 articles and trials, with a focus on the diagnosis and management of MSCC. Key findings from these included: diagnostically, Magnetic Resonance Imaging (MRI) is the hallmark of imaging in MSCC, with 93% sensitivity and 97% specificity [5]. Contrast-enhanced MRI allows for superior soft-tissue contrast than other modalities, thus offering better detection of metastatic infiltration and adjacent soft-tissue involvement [4]. From an interventional perspective, radiotherapy remains a central tenet of MSCC management. Decisions around the course of radiotherapy are driven by patient prognosis. Notably, single-dose and short-course radiotherapy have been found to be non-inferior to long-course radiotherapy regarding symptom control [9, 10], thus allowing a more convenient option for patients being approached with palliative intent.

Advancements in radiotherapeutic techniques, including novel approaches such as stereotactic body radiosurgery, have allowed for greater local tumor control and efficacy against radioresistant tumors [11]. Surgical advancements, including minimally invasive spine surgery and kyphoplasty, have provided additional solutions for mechanical instability and painful vertebral compression fractures [12]. Bone-modifying agents, such as zoledronic acid, have been effective in reducing the incidence and recurrence of skeletal-related events, and thus could be considered as adjuvants for postoperative management [13]. Early mobilization following interventions and involvement of multidisciplinary teams to streamline outcomes for patients has also been found to be effective post-operative management strategies [14, 15].

Summarizing the current advancement in MSCC, this review highlights the critical role of appropriate diagnosis and therapy, and aims to refine treatment protocols, including post-operative rehabilitation, pain management, and prevention of further skeletal-related events. It advocates for a personalized approach that balances efficacy, safety, and quality of life for patients

Methodology

A literature search strategy was employed to identify relevant studies, focusing on the topic of investigation: the current diagnosis and treatment advancements in MSCC and its post-intervention care. 604 articles were selected from PubMed, using the search string "Spinal Cord Compression"[Mesh] OR "The Current Advancements in The Treatment of Metastatic spinal cord Compression" OR "diagnosis of Metastatic spinal cord Compression" OR "post intervention care of Metastatic spinal cord Compression.” After the screening process and a comprehensive round of data extractions, the authors selected 31 articles, based on the inclusion criteria: only human studies in English-language with emphasis on the adult population, published within the past 10 years (2015-2025). The review was designed to be a narrative review as opposed to a systematic review, to allow for a broader exploration of emerging trends, expert opinions, and evolving clinical practices that may not yet be captured in structured evidence synthesis. Our review combined data from clinical trials, meta-analyses, randomized controlled trials, and systematic reviews, some of which are listed within Table 1.

**Table 1:** Summary of pertinent clinical research parsed for study MESCC: metastatic epidural spinal cord compression; MSCC: malignant spinal cord compression; SRS: stereotactic radiosurgery; SBRT: stereotactic body radiation therapy

Title	Author(s)	Year of Publication	Study Type	Sample Size	Objective
Predicting Neurologic Recovery after Surgery in Patients with Deficits Secondary to MESCC: Systematic Review	Laufer I et al [1]	2016	Systematic Review	41 members of AO Spine Knowledge Forum Tumor	Determine factors associated with neurologic improvement in patients with MESCC who undergo surgery.
Recent advances and new discoveries in the pipeline of the treatment of primary spinal tumors and spinal metastases: a scoping review	Furlan JC et al [2]	2021	Clinical Study Review	N/A	Summarizes current knowledge and future directions in spinal neoplasms treatment.
Comprehensive Insights into Metastasis-Associated Spinal Cord Compression	Vavourakis M et al [3]	2024	Systematic Review	N/A	Provide a comprehensive analysis of MSCC pathophysiology, diagnosis, treatment, and prognosis.
Imaging of metastatic epidural spinal cord compression	Bai J et al [4]	2022	Narrative Review	N/A	Update on imaging techniques for MSCC diagnosis, differential diagnosis, and management.
State-of-the-Art Imaging Techniques in Metastatic Spinal Cord Compression	Kuah T et al [5]	2022	Narrative Review	N/A	Assess strengths/limitations of imaging in MSCC and explore deep learning advances.
Spinal Metastases of the Vertebrae: Three Main Categories of Pain	Van den Brande R et al [6]	2024	Narrative Review	N/A	Understand pathophysiology and symptoms of spinal metastases and treatment options.
Epidural Spinal Cord Compression as the Presenting Manifestation of Acute Myeloid Leukemia: A Case Report and Literature Review	Fujikawa T et al [7]	2023	Literature Review	23 cases of Acute Myeloid Leukemia (AML)	Report a rare case of MSCC due to AML and emphasize early intervention.
Direct decompressive surgical resection in the treatment of spinal cord compression caused by metastatic cancer: a randomised trial	Patchell R et al [8]	2005	Clinical Trial	123 patients	Compare surgery + radiotherapy vs radiotherapy alone in MSCC.
Interventions for the treatment of metastatic extradural spinal cord compression in adults	George R et al [9]	2015	Systematic Review	7 RCTs, 876 participants	Evaluate efficacy and safety of surgery, radiotherapy, and corticosteroids for MSCC.
Conventional Radiotherapy and Stereotactic Radiosurgery in the Management of Metastatic Spine Disease	Zhang H-r et al [10]	2020	Narrative Review	N/A	Compare conventional radiotherapy and SRS for spinal metastases.
Stereotactic Radiosurgery for Postoperative Spine Malignancy: A Systematic Review and International Stereotactic Radiosurgery Society Practice Guidelines	Faruqi S et al [11]	2022	Systematic Review	461 patients	Assess safety and efficacy of postoperative spine SBRT and provide guidelines.
Effect of Longer-Interval vs Standard Dosing of Zoledronic Acid on Skeletal Events in Patients With Bone Metastases: A Randomized Clinical Trial	Himelstein A et al [13]	2017	Randomized Clinical Trial	1822 patients	Compare dosing intervals of zoledronic acid in patients with bone metastases.

## Review

Advancements in diagnostics

Initial Presentation and Imaging Tools

The most common initial symptoms of MSCC include low back pain, weakness in the lower limbs, and, in severe cases, changes in bladder and intestinal function. Although these signs are not specific, they must be evaluated urgently, especially in patients with a history of cancer [5, 16, 17].

**Figure 1:**
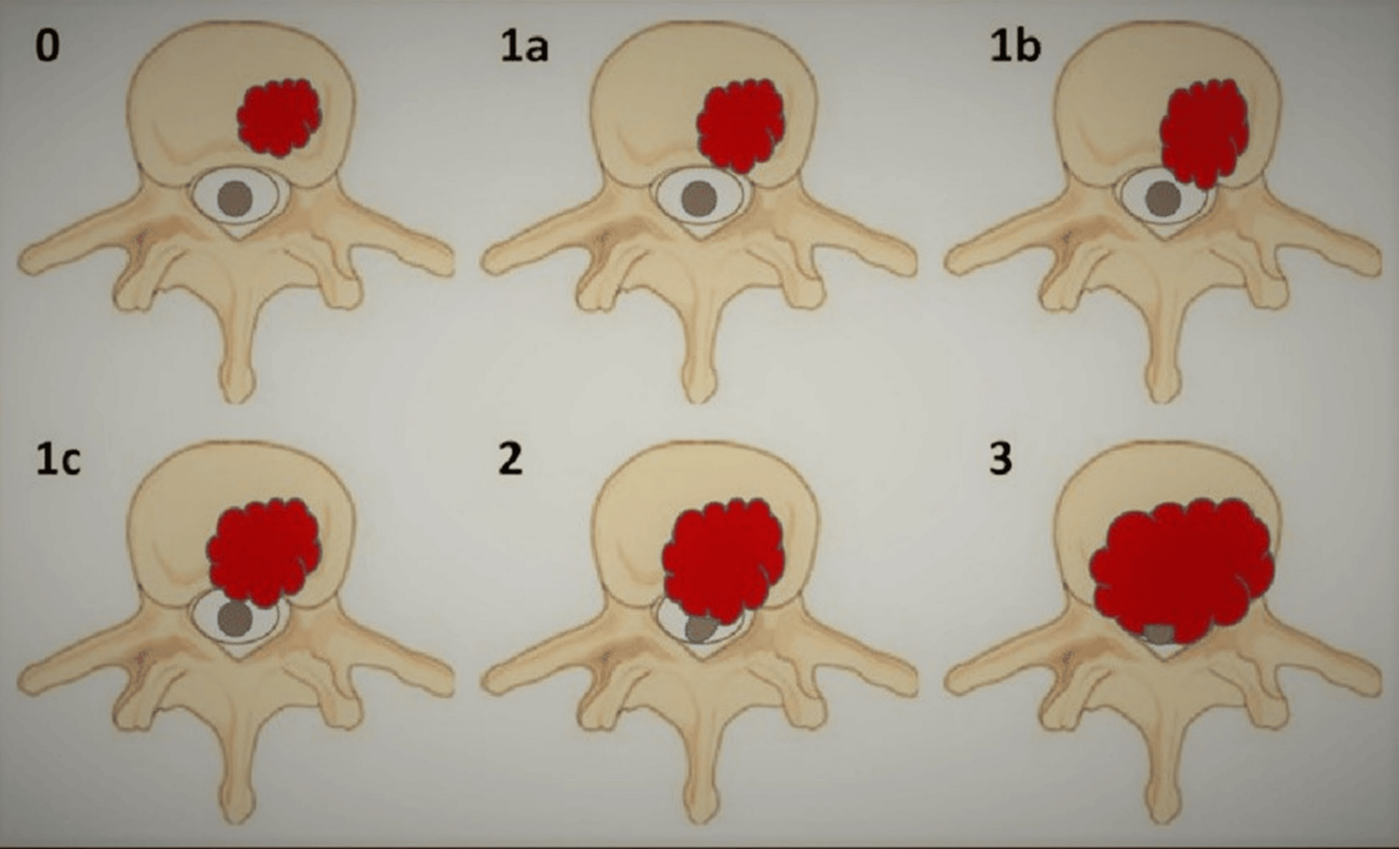
Grading of Epidural Spinal Cord Compression Using the Bilsky Criteria T2-weighted MRI images showing grades 0-3 of spinal cord compression. Grade 0: No deformation; Grade 1: Epidural impingement without spinal cord compression; Grade 2: Spinal cord compression with visible cerebrospinal fluid (CSF); Grade 3: Spinal cord compression with no visible CSF. Figure credit: Reproduced with permission from Zaveri et al. [18].

MRI is the gold standard for identifying spinal metastatic cord compression due to its higher sensitivity and specificity, which uses the Bilsky criteria, as shown in Figure 1, to grade the level of compression [5, 12]. T1-weighted (T1w), T2-weighted (T2w), and Short Tau Inversion Recovery (STIR) sequences are highly accurate MRI sequences for detecting spinal osseous metastases. STIR suppresses fatty marrow signals and enhances the visibility of marrow-replacing intraosseous neoplastic lesions, while the Dixon technique uses chemical shift principles for fat suppression.

CT myelograms are an alternative because they have comparable capabilities to MRI. However, when MRIs are not readily accessible, the conventional CT is favored as it allows for prompt action to improve patient outcomes, despite its lower accuracy [5, 19]. PET CT is particularly useful when there is marrow involvement.

Advanced Tools and Adjunctive Techniques

New emerging technologies, such as the application of AI to imaging and advanced techniques for metal artefact reduction, enhance diagnostic accuracy and aid in efficient treatment planning [5]. Important tools such as the Spinal Instability Neoplastic Score (SINS), and NOMS (Neurological, Oncological, Mechanical Instability, Systemic Disease), as shown in Table 2 and Table 3, have been designed to evaluate the patient's eligibility for surgery and to establish whether the aim of the treatment is curative or palliative [20, 14 ].

**Table 2:** SINS - Spinal Instability Neoplastic Score Higher scores are suggestive of spinal instability. Scores of 0-6 indicate a stable spine, while scores between 7 and 12 indicate indeterminate stability, thus requiring consultation with a spinal surgeon. Scores ≥13 are suggestive of instability and are likely to require surgical intervention. Source: Adapted with permisson from Fisher et al. [21].

Element of SINS	Score
Location	
Junctional (occiput-C2, C7-T2, T11-L1, L5-S1)	3
Mobile spine (C3-C6, L2-L4)	2
Semi-rigid (T3-T10)	1
Rigid (S2-S5)	0
Pain relief with recumbency and/or pain with movement/loading of the spine	
Yes	3
No (occasional pain but not mechanical)	1
Pain-free lesion	0
Type of bone lesion	
Lytic	2
Mixed (lytic/blastic)	1
Blastic	0
Radiographic spinal alignment	
Subluxation/translation present	4
De novo deformity (kyphosis/scoliosis)	2
Normal alignment	0
Degree of vertebral body collapse	
>50% collapse	3
<50% collapse	2
No collapse with >50% body involved	1
None of the above	0
Posterolateral involvement of the spinal elements (facet, pedicle, or costovertebral joint fracture or replacement with tumor)	
Bilateral	3
Unilateral	1
None of the above	0

**Table 3:** NOMS Decision Framework ESCC is calculated according to the ESCC scale (also known as the Bilsky scale). NOMS: Neurological, Oncological, Mechanical Instability, Systemic Disease; cEBRT: conventional external beam radiotherapy; SRS: stereotactic radiosurgery; ESCC: epidural spinal cord compression Source: Adapted with permission from Laufer et al. [22].

Neurologic	Oncologic	Mechanical	Systemic Decision	Outcome
Low-grade ESCC (0-1), no myelopathy	Radiosensitive	Stable		cEBRT
		Unstable		Stabilization followed by cEBRT
	Radioresistant	Stable		SRS
		Unstable		Stabilization followed by SRS
High-grade ESCC (2-3), ± myelopathy	Radiosensitive	Stable		cEBRT
		Unstable		Stabilization followed by cEBRT
	Radioresistant	Stable	Able to tolerate surgery	Decompression/stabilization followed by SRS
		Stable	Unable to tolerate surgery	cEBRT
		Unstable	Able to tolerate surgery	Decompression/stabilization followed by SRS
		Unstable	Unable to tolerate surgery	Stabilization followed by cEBRT

In the event of unavailability of the above imaging modalities due to financial constraints, the study of biomarkers such as indicators of bone turnover and serum and urinary levels (like type 1 collagenC-terminal telopeptide (CTx) and procollagen type 1 N-terminal propeptide (P1NP)) to identify tumors prior to or following metastasis has proven to be beneficial and offers significant predictive and prognostic value [15, 17].

To summarize, MRI remains the gold standard for diagnosis, with further enhancement in diagnosis through adjuncts such as the Bilsky criteria and modified techniques such as Dixon. Alternative modalities, such as CT-myelography and PET-CT, are used in cases with bone marrow involvement, limited access to MRI, or when there are contraindications to MRI. Breakthroughs in AI-assisted imaging and metal artefact reduction techniques have also contributed to improved diagnostic capabilities, thus allowing for more efficient and individualized treatment approaches. In addition to relatively novel tools like biomarker integration, these have provided promising adjuncts to guide predictions and prognosis, especially in resource-constrained settings.

Advancements in treatment

Radiotherapy and Radiosurgery

A comparison between conventional external beam radiotherapy (cEBRT) and stereotactic radiosurgery (SRS) showed that SRS does not provide a significant advantage in pain control. At three months, the pain response rate was 40.3% with SRS versus 57.9% with cEBRT [23]. It was evidenced that a single fraction radiotherapy scheme (8 Gy) is as effective as fractional treatments in controlling pain and maintaining mobility. However, it comes with a slight increase in the risk of local tumor recurrence [9]. More advanced techniques, such as SRS and intensity-modulated radiotherapy (IMRT), guarantee better local control of the disease with lower toxicity, making them particularly useful for radioresistant tumors or for patients who require re-irradiation [24, 5].

Surgical Approaches

Decompression surgery, combined with radiotherapy, has been shown to significantly improve walking ability (84% of patients treated surgically maintained the ability to walk compared to 57% of those treated only with radiotherapy), reduce the need for opioids, and improve bladder control. However, isolated laminectomy did not bring additional benefits compared to radiotherapy alone [9]. An increasingly used approach is separation surgery, which involves the separation of the spinal cord from the tumor mass before performing a targeted SRS. This method has shown not only an improved local control of the disease but also reduces the risks of spinal cord damage and enhances the precision and effectiveness of SRS [14, 10]. In addition, minimally invasive techniques, such as kyphoplasty, vertebroplasty and percutaneous fixation with pedicle screws, have also been shown to be effective in reducing pain, stabilizing the spine, and preserving neurological function. [12, 14, 25]

Pharmacological Interventions

High-dose corticosteroids are commonly used for rapid relief from symptoms, although they have not demonstrated a significant impact on walking ability, survival, or pain control. Their use is associated with side effects such as hyperglycemia, osteoporosis, immunosuppression, increased risk of infections, stomach ulcers, and, less frequently, hypertension. Besides, bone-modifying agents (BMAs), such as zoledronate and denosumab, have been evidenced to delay bone disease events and, in some cases, improve survival rate; specifically, zoledronate has been shown to reduce the rate of bone pathological fractures [26]. Meanwhile, new immunotherapeutic treatments, such as programmed cell death protein 1 (PD-1) inhibitors, are being studied for the potential improvement in therapeutic response in patients with multiple myeloma and spinal cord compression [27].

Combinatorial Considerations

Radiotherapy stands out as a non-invasive option due to its effectiveness in pain management and in preserving patient autonomy. Effective management of MSCC often requires the integration of multiple therapies to improve patient outcomes. When intensity-modulated radiotherapy (IMRT) is incorporated into stereotactic radiotherapy (SRT), its superiority over conventional external beam radiotherapy (cEBRT) becomes evident [24]. Local control (LC) rates with spinal stereotactic body radiation therapy (SBRT)exceed 80% to 90% at 1 to 2 years, compared to cEBRT, which achieves LC rates of 61% to 71% at 1 year [28]. However, precise guidelines regarding dosing, targeting, and patient selection criteria have yet to be defined.

It has also been observed that combining radiotherapy with decompression surgery enhances the outcomes of spinal radiosurgery (SRS). For instance, the ambulatory rate increased from 68% before treatment to 84% after treatment [11]. Nonetheless, cEBRT alone remains an excellent option for pain control. Among 50% to 80% of patients with distressing symptoms from bone metastases, conventional radiotherapy provided effective palliative care with minimal side effects two years after a single 8 Gy fraction [24]. These findings underscore the importance of carefully assessing each individual case to determine whether a single modality or a combination of therapeutic approaches is most appropriate.

Factors like the presence of mechanical instability can play a key role in selecting the most appropriate choice and obtaining the ideal outcome, which should involve an increase in the patient's autonomy and mobility and a decrease in pain or impaired functionality. While the use of corticosteroids was associated with crucial short-term symptom relief, particularly in reducing inflammation and alleviating neurological deficits, the impact of drug therapy on long-term mobility and survival outcomes was shown to be very limited [9]. Nevertheless, it is still a valuable option for patients presenting with first symptoms and requiring immediate pain alleviation. However, notable side effects should emphasize the carefulness of pharmacological prescription and use. Newer immunotherapies like PD-1 inhibitors could provide new insight for non-invasive methods in specific cases. Lastly, careful selection of the treatments presented should be done based on the individual case, along with the correct diagnosis.

Post-intervention care

A Multidisciplinary Recovery Model

Creating a personalized treatment plan leads to a faster and more positive outcome, achieved through a multidisciplinary approach involving oncologists, radiation therapists, neurosurgeons, physiotherapists, and pain specialists [15]. Early recovery and effective rehabilitation are crucial for improving a patient's quality of life and overall outcome post-surgery [1, 29, 30].

Evidence-Based Rehabilitation

Evidence suggests that combining surgery and radiotherapy with pharmacotherapy reduces opioid dependence, optimizes pain control, and improves rehabilitation and reintegration into daily life [25, 23]. A poor prognosis is linked to widespread metastases and severe spinal instability, making timely surgery and radiotherapy essential for better outcomes [23]. Spinal instability can be assessed using the Spinal Instability Neoplastic Score (SINS), where five imaging parameters help guide surgical consultation (≥7 suggests the need for intervention). It is also used alongside the Epidural Spinal Cord Compression (ESCC) scale for post-treatment monitoring [4, 20, 14].

Despite challenges and the need for further research, future MSCC treatment is expected to benefit from minimally invasive approaches and emerging techniques like da Vinci Robots (Intuitive Surgical, Sunnyvale, USA) and Laser Interstitial Thermotherapy, leading to improved prognosis through significantly lower injury and better quality of life [20].

Significance

Multiple advancements in oncological management across the world have resulted in rising survival rates for cancer patients globally [31]. While this marks a commendable achievement for the field of oncology, it has also directly contributed to an increase in the risk of secondary outcomes and incidence of late-stage metastases, thereby directly increasing the risk of skeletal-related events (SRE) such as MSCC [32].** **This assessment highlights the importance of continuous revision and training for healthcare providers to ensure timely diagnosis and appropriate treatment in order to preserve neurological function and improve the quality of life for patients.

The betterment of outcomes has been led by a shift towards precision medicine, with the aid of AI-enhanced diagnostic models, improved therapeutic interventions, and an added focus on a multidisciplinary approach. AI-driven diagnostic models have been shown to help with management and avoiding delayed diagnosis, with studies emphasizing outcomes similar to those of subspecialist radiologists and clinical specialists [33, 34].

From an interventional perspective, minimally invasive surgery and robotic-assisted surgery have been shown to be key evolutionary aspects of spinal surgery and tumor management. From the advent of Mazor Spine Assist (Medtronic, Minneapolis, USA), the first FDA-approved robot licensed for pedicle screw replacement in 2004, the technique has grown to allow significantly increased accuracy and reduced radiation exposure in decompressions, dural closure, and osteotomies, thus leading to improved outcomes in terms of reduced blood loss during surgery and shorter recovery times [35]. Therapeutic modalities such as SRS have been shown to have 80% pain response rates, thus allowing patients to have improved mobility and overall well-being, all while reducing the opioid dependence burden [36].

A multidisciplinary approach is essential to the correct identification of the symptomatology of MSCC patients, together with collaboration among oncologists, surgeons, radiation therapists, and rehabilitation specialists, and a careful selection of the available treatments depending on the individual case can significantly enhance patient outcomes including improving quality of life, survival rate and limiting undesired results. Improved collaboration leads to better decision-making, allowing for more significant differentiation between surgical and non-surgical approaches and achieving a better balance between curative and palliative goals.

Moving ahead, MSCC management is likely to be refined by ongoing research into AI-driven diagnostic tools, robotic-assisted surgeries, and novel systemic therapies aimed at decreasing both the incidence and post-operative recurrence of MSCC. At the systems level, improvements in the field are tied to standardizing guidelines, improving access to advanced imaging and therapeutics, and ensuring reduced disparities in care delivery in resource-constrained environments.

Addressing limitations

This narrative review has potential limitations, including its selective criteria and the inclusion of only English-language articles that were published within the past decade. This selection bias may favor studies from certain geographical locations, making it applicable for a narrower population of the general public and omitting long-term cohort data, which could have provided valuable insight into treatment outcomes.

Moreover, the review may be restricted for the generalizability of findings across a wide range of populations because its main focus is MSCC and does not account fully for variability in cancer behavior. Different cancer subtypes exhibit distinct levels of aggressiveness, metastatic burden, and therapeutic responses.

Although similar interventions are used for curative and palliative treatments, a notable limitation is the absence of clear, standardized measures to differentiate between treatment intent strategies. The lack of criteria places the decision-making in the hands of the clinician, who, if risk-averse, may favor palliative approaches over curative strategies, which may unintentionally bias the literature.

## Conclusions

In conclusion, the integration of advanced diagnostic modalities, from AI-enhanced MRI techniques to biomarker-driven approaches, combined with precision surgical and radiotherapeutic interventions, is revolutionizing the management of MSCC. These innovations not only improve patient outcomes and quality of life but also set the stage for a new era of personalized, multidisciplinary approaches in oncology and other specialties. Ultimately, sustained research and collaboration are imperative to ensure that every patient receives timely and effective treatment, transforming the landscape of MSCC management.
